# Neurodevelopmental differences in task-evoked number network connectivity: Comparing symbolic and nonsymbolic number discrimination in children and adults

**DOI:** 10.1016/j.dcn.2022.101159

**Published:** 2022-10-04

**Authors:** Mikael Skagenholt, Kenny Skagerlund, Ulf Träff

**Affiliations:** a Department of Behavioral Sciences and Learning, Linköping University, Linköping, Sweden; b Department of Management and Engineering, JEDI-Lab, Linköping University, Linköping, Sweden; c Center for Social and Affective Neuroscience (CSAN), Linköping University, Linköping, Sweden

**Keywords:** TCM, Number processing, Development, FcMRI, Numerical cognition, Connectivity

## Abstract

Numerical cognition can take place in multiple representational formats, such as Arabic digits (e.g., 1), verbal number words (e.g., “two”), and nonsymbolic (e.g., •••) numerical magnitude. Basic numerical discrimination abilities are key factors underlying the development of arithmetic abilities, acting as an important developmental precursor of adult-level numeracy. While prior research has begun to detail the neural correlates associated with basic numerical discrimination skills in different representational formats, the interactions between functional neural circuits are less understood. A growing body of evidence suggests that the functional networks recruited by number discrimination tasks differ between children and adults, which may provide valuable insights into the development of numerical cognition. To this end, we posed two questions: how do the interactions between functional circuits associated with number processing differ in children and adults? Are differences in functional network connectivity modulated by numerical representational codes? A theoretically motivated 22 ROI analysis indicated significant functional connectivity differences between children and adults across all three codes. Adults demonstrated sparser and more consistent connectivity patterns across codes, indicative of developmental domain-specialization for number processing. Although neural activity in children and adults is similar, the functional connectivity supporting number processing appears subject to substantial developmental maturation effects.

## Highlights

• Identified developmental differences in symbolic and nonsymbolic number processing.
• Adults’ functional connectivity indicative of developmental ANS estrangement.
• Greater connectivity overlap between symbolic (Arabic, verbal) number in adults.
• Children’s functional connectivity indicates domain-general effortful processing.
• Number symbols increasingly processed by retrieval than mapping over development.

## 1 Introduction

Numerical cognition ranges from biologically predisposed and preverbal capacities for the approximate discrimination of numerical magnitude (e.g., Núñez, 2017), to the calculation of complex arithmetic leveraging exact symbolic representations of number (e.g., Arabic digits, number words). Number symbols have traditionally been argued to gain their semantic content by comparison and mapping onto the so-called approximate number system (ANS; Odic and Starr, 2018), entailing that the ability to distinguish 4 (four) as greater than 2 (two) hinges on similar core-cognitive mechanisms employed when discriminating •••• as a greater quantity than •• (4 > 2). Simultaneous activation of representations bootstraps symbolic referents to the corresponding nonsymbolic quantity, in line with the triple code model (TCM; Dehaene, 1992). This results in a common amodal (i.e., notation-independent) and abstract representation of numerical magnitude along a mental number line (e.g., Piazza et al., 2004; Nieder, 2016), subserved primarily by numerosity-coding neurons in the intraparietal sulcus (IPS; Dehaene et al., 2003). The predictive power of ANS-based measures for symbolic number processing abilities has recently been questioned (e.g., Marinova et al., 2018), suggesting that number symbols may rather gain their semantic content from other number symbols (e.g., by understanding symbolic numbers’ associative relationships in the count-list). For instance, performance on symbolic numerical order verification tasks (leveraging knowledge of the count-list) becomes a better predictor of applied arithmetic ability than nonsymbolic quantity discrimination tasks over the course of middle school (Lyons et al., 2014). In adults, overlapping brain activation for nonsymbolic numerical order and magnitude processing is greater than the overlap between symbolic and nonsymbolic order processing, suggesting that symbolic numbers are processed independently of the ANS (e.g., Lyons and Beilock, 2013). Children’s neural correlates of symbolic numerical order processing appear less consistent, overlapping with regions attributed to both symbolic and nonsymbolic numerical magnitude processing (e.g., superior, inferior, and middle frontal gyri, insula; Kucian et al., 2011). Symbolic numerical cognition may therefore progressively dissociate from nonsymbolic number processing over developmental time, in line with the *symbolic estrangement hypothesis* (e.g., Lyons et al., 2012). Given that symbolic number processing requires education and experience, as opposed to the core-cognitive ability for nonsymbolic number discrimination (Núñez, 2017), we reasoned that developmental differences may be informative for studying representational differences in numerical cognition. Correspondingly, recent research indicates “growing evidence to suggest that functional circuits engaged by children are not the same as those engaged by adults” (Iuculano et al., 2018, p. 320). To this end, two research questions emerged: how do the interactions between functional neural circuits engaged by children and adults during number discrimination differ, and are these differences modulated by different numerical representational codes?

This study builds on prior functional magnetic resonance imaging (fMRI) research from our lab (Skagenholt et al., 2021), examining middle-school-aged children’s and young adults’ neural activity elicited by Arabic digit, number word, and nonsymbolic numerical dot array discrimination tasks (i.e., selecting the numerically greater alternative). Acting as a direct empirical validation of previous meta-analytic investigations into the predictions of the TCM (e.g., Arsalidou and Taylor, 2011; Arsalidou et al., 2018), the results indicated minor neural activity differences between the two age-groups across tasks. Primarily, adults uniquely recruited regions typically attributed to domain-general decision-making, such as the anterior cingulate cortex (ACC), across all tasks. Although the role of the ACC is rather elusive given its broad involvement across cognitive domains (e.g., Heilbronner and Hayden, 2016), the region (together with the dorsolateral prefrontal cortex and IFG) has been characterized as part of a cognitive control network supporting the semantic retrieval of symbolic numbers (Kersey and Cantlon, 2017). This partially aligns with unique activity previously found during adult verbal number discrimination (ACC, IFG, and middle frontal gyrus; Skagenholt et al., 2021). However, no speed–accuracy tradeoff was observed in adults across all tasks, suggestive of developmental performance gains that should also be observable at the neural level. We hypothesized that these relatively minor activity differences indicate that 11-year-old children have reached a near-mature functional–organizational state with regards to the regions recruited for number discrimination, and that remaining developmental differences may rather emerge as distinct patterns of functional connectivity between these regions. Emerson and Cantlon (2012) similarly found that, during a cross-format (digits and dot arrays) number matching task, adults showed greater frontoparietal functional connectivity despite exhibiting equivalent neural activity as a matched child sample. The current study extended this approach to examine if frontoparietal network connectivity, across 22 theoretically motivated regions of interest (ROI; see Figure and Table 1), is modulated by age and numerical representational formats (i.e., Arabic, verbal, and nonsymbolic number representations).

**Table 1:** Selected neural regions of interest.

Region of interest	Seeds	Node	Peak MNI (left; right)
Intraparietal sulcus (IPS)	2	179, 43	−36, −39, 48; 32, −61, 49
Supramarginal gyrus (SMG)	2	181, 46	−60, −26, 22; 58, −29, 20
Precentral gyrus (PreCG)	2	165, 27	−46, 0, 49; 49, −5, 48
Inferior frontal gyrus (IFG)	2	156, 21	−53, 18, 11; 55, 10, 22
Anterior insula (AI)	2	168, 35	−39, 2, 10; 41, 4, 7
Fusiform gyrus (FG)	2	206, 72	−43, −70, −14; 21, −64, −9
Dorsolateral prefrontal cortex (MFG)	2	146, 11	−27, 34, 36; 38, 35, 31
Hippocampus (HC)	2	232, 94	−36, −25, −15; 36, −15, −18
Cerebellum lobule VIIa/VI (CB)	2	238, 113	−37, −53, −31; 37, −57, −33
L Superior frontal gyrus (SFG/FEF)	1	150	−5, 18, 46
L Angular gyrus (AG)	1	182	−42, −66, 42
L Middle temporal gyrus (MTG)	1	192	−58, −48, 5
L Anterior cingulate cortex (ACC)	1	219	−6, 34, 26

Seeds indicate number of seed regions per ROI. Node (left, right) refers to associated ROI parcellation numbers in the Shen 268 node atlas (Shen et al., 2013).

Three sources were used to select the included ROIs. A meta-analysis of regions associated with number processing in healthy and impaired participants (Faye et al., 2019) detailed a numerical cognition circuit common to both symbolic and nonsymbolic number processing tasks, implicating the bilateral IPS, supramarginal, inferior frontal, as well as angular gyri (SMG, IFG, AG), dorsolateral prefrontal cortex (DLPFC), superior frontal language area, and middle temporal gyrus (MTG). A systems neuroscience review of numerical and mathematical cognition in typical and atypical development (Iuculano et al., 2018) detailed sixteen neural regions prevalent throughout the literature. Beyond the numerical cognition circuit (Faye et al., 2019), the inclusion of the fusiform gyrus (FG) and hippocampus (HC) is particularly relevant. The right FG preferentially responds to Arabic digits over other symbolic stimuli, leading the region to be considered a *visual number form area* akin to the left FG’s *visual word form area*, with similar preference for words over word-like control stimuli (e.g., Hannagan et al., 2015; Grotheer et al., 2016). Although the HC is mainly discussed in the context of mathematical learning and arithmetic, it stands to reason that the same developmental increase in hippocampal–neocortical connectivity occurring as children transition from counting to retrieval of arithmetic facts (Qin et al., 2014) applies to the retrieval of basic number facts (e.g., [4 = four = ••••] > [2 = two = ••]). Following the symbolic estrangement hypothesis, we predicted that adults perceive and manipulate symbolic number in an associative manner, expecting greater functional connectivity between the left IPS (specialized for symbolic processing over development; Vogel et al., 2015), left and right FG (number word and digit identification respectively), as well as memory retrieval regions (SMG, AG, MTG, HC; Iuculano et al., 2018). Finally, ROI selection was informed by a review of functional and structural brain connectivity in numerical cognition (Moeller et al., 2015). Nine ROIs for number discrimination were consistently identified: the bilateral IPS, precentral gyrus, and prefrontal cortex; the left SMG and pre-supplementary motor area; and right superior parietal lobe. The regions included in the current study therefore correspond well to a frontoparietal network of regions consistently associated with numerical cognition (e.g., Fias et al., 2013), including domain-specific number identification and manipulation mechanisms (e.g., FG, IPS) as well as domain-general abilities such as working memory and cognitive control (e.g., premotor cortex and DLPFC), attention (e.g., insula, ventrolateral cortex), and semantic memory and language abilities (e.g., AG, SMG, MTG, HC). Three less common regions, identified in our previous univariate results, were included for exploratory purposes: the bilateral cerebellar lobule VI/VIIa (cf. Vandervert, 2017) and left ACC.

Children generally rely more on frontal brain regions (e.g., right IFG) for symbolic number processing, indicating a frontal-to-parietal migration over developmental time (e.g., Ansari et al., 2005; Kaufmann et al., 2006). With increased attentional and working memory demands, attributable to immature symbol-mapping and retrieval capacities, we expected greater functional connectivity between the numerical quantity-processing right (as opposed to symbolic number-specialized left) IPS, cognitive control systems (SFG, MFG) as well as salience and attention control processes (IFG, insula) across tasks (Iuculano et al., 2018). Note that this prediction is informed by neural activity, whereas connectivity results suggest greater PFC–IPS connectivity in adults (Emerson and Cantlon, 2012). As prior results (Skagenholt et al., 2021) indicated no child-specific increases in neural activity, resembling results from Emerson and Cantlon (2012), a similar age-dependent connectivity increase is possible. The developmental frontoparietal shift, interpreted as decreased reliance on cognitive control, attention, and working memory (Ansari, 2008), is nonetheless well-established in the literature (e.g., Rivera et al., 2005). Child numerical deficit intervention studies could reconciliate these positions. Frontoparietal hyperconnectivity for basic number processing has been observed to decrease with training and experience (e.g., Jolles et al., 2016; Michels et al., 2018). However, arithmetic training shows general activation increases in memory-related regions (e.g., AG, MTG) for simple multiplication problems, whereas training on complex problems increases frontoparietal activity in math-deficient children (Soltanlou et al., 2022). It could be argued that the need for (primarily frontal) domain-general compensatory cognitive mechanisms decreases with age and experience, when basic number discrimination (akin to simple rote multiplication) becomes increasingly retrieval-based (Iuculano et al., 2018). The results of the current study may help to disentangle these hypotheses, beyond providing an overview of developmental and representation-specific functional connectivity differences for numerical cognition.

## 2 Methods

### 2.1 Participants

A total of ninety-one healthy, right-handed participants participated across two studies: one featuring child participants (*N* = 39) and one with young adult participants (*N* = 51). Both studies were conducted using the same MRI scanner, hardware, protocol, as well as experimental tasks and analysis pipeline. None of the participants had any clinically documented or self-reported health conditions, or deficits in mathematical or general cognitive abilities. Participating families in the child study were not paid; adult participants were paid approximately $60. Both studies were approved by the Regional Ethical Review Board in Linköping, Sweden (child study approval reference: 2018/513–32; adult study approval reference: 2017/103–31). Written informed consent was obtained from participating adults and legal guardians prior to participation. Participating children were additionally asked for verbal consent. Following data quality control and denoising, a total of 88 participants remained for further analysis (37 children, *mean age* = 11.41, *SD* = 0.55; 51 adults, *mean age* = 23.36; *SD* = 2.86). Gender distributions for the child study were 12 girls and 25 boys, and the adult study featured 29 females and 22 males.

### 2.2 Neuroimaging tasks

Child participants performed an hour-long mock scanner practice session prior to participation, familiarizing them with the environment (including MRI sounds from SimFX; Psychology Software Tools, Sharpsburg, PA, USA), tasks, and effects of motion (MoTrak software with associated motion sensor; Psychology Software Tools, Sharpsburg, PA, USA). Three trials of each task were repeated until participants reached full accuracy, to ensure task comprehension.

Three Echo Planar Imaging (EPI) blood-oxygen-level-dependent (BOLD) sequence runs were administered over the course of the fMRI scanning session, lasting approximately 62 min. Tasks were administered, in a fixed order, using an alternating blocked design meant to minimize time between recurring instances of similar trials (Henson, 2007). Task blocks were interspersed with 12 s resting periods meant to return the hemodynamic response signal to baseline level. For each EPI run, six tasks were administered (for tasks beyond the scope of this paper, see Skagenholt et al., 2018). Participants performed, in order: Arabic digit comparison, verbal number comparison, nonsymbolic magnitude comparison, and a basic decision-making control task. Each experimental task was administered twice within a run, such that the first iteration of 14 trials consisted of “easy” far-distance trials (e.g., 2 vs 6) and the second iteration consisted of “hard” near-distance trials (e.g., 8 vs 9), in line with the numerical distance effect (see Fig. 1 in Skagenholt et al., 2021). Each task featured 84 trials in total (14 trials × 2 distances × 3 runs). Each trial was preceded by a 500 ms fixation cue, followed by stimulus presentation lasting for 2000 ms, and concluded with a response cue (“?”) lasting for 1500 ms. Participants recorded their response, during the response cue, using buttons placed beneath their right index (for leftward responses) and middle (for rightward responses) fingers (Lumia response pad; Cedrus Corporation, San Pedro, CA, USA). SuperLab 5 (Cedrus Corporation, San Pedro, CA, USA) was used to administer trials, which were presented in a pair of VisuaStimDigital MRI-safe video goggles (Resonance Technology Inc., Northridge, CA, USA).

**Fig. 1:**
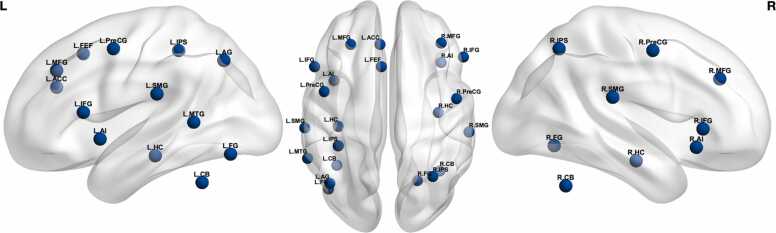
Regions of interest (ROI) for functional connectivity analysis.

#### 2.2.1 Arabic digit comparison

Stimuli were two Arabic digits, placed horizontally on the left and right side of the screen. Participants were instructed to select the numerically larger digit by pressing the corresponding button on the left (index finger) or right-hand (middle finger) side. The first iteration of trials in a run featured far-distance trials (numerical distance of 4–5; e.g., 5 vs 9) whereas the second iteration featured near-distance trials (numerical distance of 1–2; e.g., 2 vs 3).

#### 2.2.2 Verbal number comparison

Similar to the Arabic digit comparison task, stimuli were two number words placed horizontally across the screen. Participants were instructed to select the numerically larger number word by pressing the corresponding left (index finger) or right-lateralized (middle finger) button on the response pad. Identical numerical distances for far and near-distance trials were used (i.e., 4–5 and 1–2; e.g., five vs nine and two vs three), occurring in the same iterations as described above.

#### 2.2.3 Nonsymbolic magnitude comparison

Dot arrays, consisting of 8–26 dots each (to discourage enumeration; Trick and Pylyshyn, 1994), were created using Panamath (version 1.22; Halberda et al., 2008). Two arrays were simultaneously presented, requiring participants to select the most numerous array to the left (index finger) or right-hand (middle finger) side of the screen. To control for visuospatial extent cues that may confound nonsymbolic magnitude discrimination, cumulative surface area and numerosity was matched in half of all trials. In line with the numerical distance effect, the far-distance iteration of trials was represented by a ratio of 1:2 (e.g., 8 vs 16 dots). The near-distance iteration was represented by a ratio of 4:3 (e.g., 14 vs 17 dots).

#### 2.2.4 Letter case discrimination control task

To control for functional connectivity patterns not directly attributable to number processing (i.e., non-numerical decision-making processes), task-evoked functional connectivity elicited by a basic decision-making task was included as a covariate of no interest in the analyses. Participants were presented with two letters placed horizontally on the screen, one upper- and one lowercase (e.g., “a” and “H”), and instructed to select the “larger” (i.e., uppercase) alternative. Stimulus presentation and response requirements were identical to the experimental tasks.

### 2.3 fMRI data acquisition

The fMRI studies were conducted at Linköping University’s Center for Medical Imaging and Visualization (CMIV). A Siemens Magnetom Prisma 3.0 T MRI scanner was used for data acquisition, featuring a twenty-channel head coil. Prior to experimental trial runs, high-resolution structural scans were acquired using a T1-weighted sequence (208 slices, slice thickness = 0.9 mm^3^, TR = 2300 ms, TE = 2.36 ms, flip = 8°). EPI pulse runs were performed using three T2 * -weighted BOLD-sensitive sequences to acquire whole-brain functional scans (48 slices, slice thickness = 3.0 mm^3^, TR = 1340 ms, TE = 30 ms, flip = 69°).

### 2.4 fMRI data preprocessing

Preprocessing was performed in the CONN toolbox (version 20.b; Whitfield-Gabrieli and Nieto-Castanon, 2012) for SPM12 (Wellcome Department of Cognitive Neurology, London, UK). The default pipeline and parameters in the toolbox were used, including functional realignment and unwarping, outlier identification (framewise displacement > 0.9 mm or global BOLD signal changes > 5 *SD*), direct segmentation and normalization into standard MNI space, and functional smoothing (6 mm full-width-at-half-maximum Gaussian kernel). No slice-timing correction was performed. Data were denoised with a band-pass filter (0.008–0.09 Hz) and linearly detrended. No despiking was performed. Two participants were excluded as more than 20% of overall volumes were flagged as outliers.

### 2.5 fMRI data analysis

ROI-based functional connectivity analysis was performed using the CONN toolbox. First-level, subject specific connectivity matrices were computed based on the Shen 268 node parcellation atlas (Shen et al., 2013). Twenty-two regions of interest (see Table 1) were selected in accordance with commonly reported MNI coordinates across three meta-analysis studies of number processing (Faye et al., 2019, Iuculano et al., 2018, Moeller et al., 2015), controlled for overlap with the frontoparietal number network (e.g., Fias et al., 2013), and verified with the probabilistic cytoarchitectonic atlas in SPM Anatomy Toolbox (Eickhoff et al., 2005). Second-level analyses were conducted using a parametric multivariate statistics approach, as to target the contributions of individual ROIs (i.e., “alternative settings for ROI-based inferences, parametric multivariate statistics” in the CONN toolbox). An FDR-corrected ROI-level *p*-value (proportion of false discoveries among ROIs with similar effects; MVPA omnibus test) was used with a threshold of *p*_FDR_ < 0.05. The connection (height) threshold of *p* < .01 (uncorrected) was used to investigate individual connections for each ROI. A between-subjects contrast was used to identify differences in functional connectivity attributable to participant age (i.e., [0 1]), whereas the main effect of each numerical code–controlled for basic decision-making ability–was established using a between-conditions contrast (i.e., [1 0]). Note that trials with both correct and incorrect responses were included in the analyses, given the use of a block design.

## 3 Results

For an overview of behavioral results (including response times and accuracies associated with experimental tasks) as well as brain activation analyses, see Skagenholt et al. (2021).

### 3.1 Arabic digit comparison

**Fig. 2:**
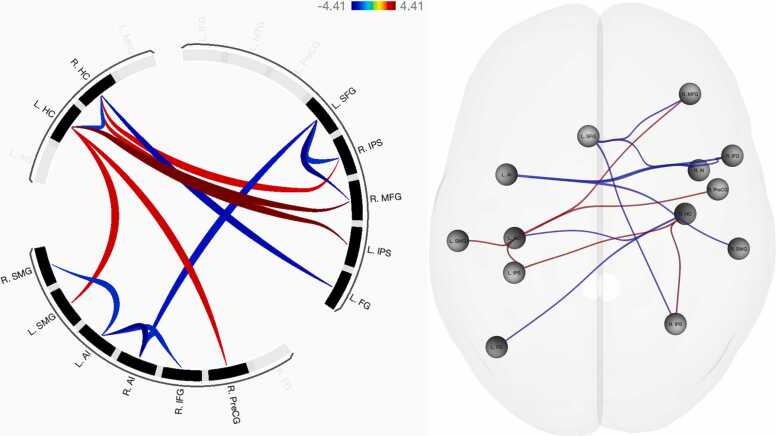
Functional connectivity differences associated with Arabic digit comparison, controlled for general decision-making ability. Blue: increased FC negatively associated with age; red: increased FC positively associated with age.

Functional connectivity patterns were observed in six seed regions (see Table 2): the left superior frontal gyrus, hippocampus, and anterior insula; as well as the right middle frontal gyrus, anterior insula, and hippocampus. See Fig. 2 for an overview of functional connectivity results.

**Table 2:** Functional connectivity patterns associated with Arabic digit comparison.

Seed region	*F* (5, 83)	*p*_FDR_	Age group	Target region	*T* (87)	*p*_unc_
L SFG	10.55	< 0.001	Child	R MFG	-4.06	< 0.001
				R Anterior insula	-3.09	0.003
				R IPS	-2.93	0.004
L Hippocampus	8.60	< 0.001	Adult	R MFG	4.41	< 0.001
				L IPS	4.39	< 0.001
				R Precentral gyrus	3.29	0.001
				L SMG	2.38	0.006
			Child	R Hippocampus	-2.78	0.007
L Anterior insula	7.01	< 0.001	Child	R Anterior insula	-4.23	< 0.001
				R IFG	-2.99	0.004
				R SMG	-2.95	0.004
R MFG	5.71	0.001	Adult	L Hippocampus	4.41	< 0.001
			Child	L SFG	-4.06	< 0.001
R Anterior insula	4.72	0.003	Child	L Anterior insula	-4.23	< 0.001
				L SFG	-3.09	0.003
				R IFG	-2.95	0.004
R Hippocampus	4.46	0.004	Adult	R IPS	2.80	0.006
				L IPS	2.77	0.007
			Child	L Fusiform gyrus	-3.63	< 0.001
				L Hippocampus	-2.78	0.007

Note: signs (-) indicate increases in functional connectivity concordant with age, such that negative signs indicate child-specific connectivity patterns and (implicit) positive signs indicate adult-specific connectivity. *F*-values and associated FDR-corrected *p*-values indicate ROI cluster-level effects. Height (connection-level) threshold: *p*_uncorrected_ < 0.01.

### 3.2 Verbal number comparison

**Fig. 3:**
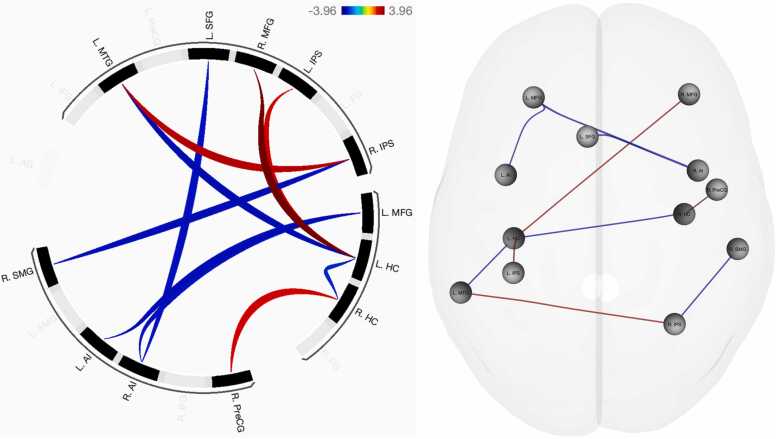
Functional connectivity differences associated with verbal number comparison, controlled for general decision-making ability. Blue: increased FC negatively associated with age; red: increased FC positively associated with age.

Functional connectivity differences between child and adult participants were observed in eight seed regions (see Table 3): the left hippocampus, superior frontal gyrus, intraparietal sulcus, and anterior insula; as well as the right intraparietal sulcus, hippocampus, anterior insula, and middle frontal gyrus. See Fig. 3 for an overview of functional connectivity results.

**Table 3:** Functional connectivity patterns associated with verbal number comparison.

Seed region	*F* (5, 83)	*p*_FDR_	Age group	Target region	*T* (87)	*p*_unc_
L Hippocampus	6.05	0.001	Adult	R MFG	3.96	< 0.001
				L IPS	2.80	0.006
			Child	L MTG	-3.20	0.002
				R Hippocampus	-2.70	0.008
R IPS	4.70	0.007	Adult	L MTG	3.36	0.001
			Child	R SMG	-3.16	0.002
L SFG	3.73	0.027	Child	R Anterior insula	-2.99	0.004
R Hippocampus	3.46	0.032	Adult	R Precentral gyrus	2.87	0.005
			Child	L Hippocampus	-2.70	0.008
L IPS	3.25	0.032	Adult	L Hippocampus	2.80	0.006
L Anterior insula	3.15	0.032	Child	L MFG	-3.18	0.002
R Anterior insula	3.15	0.032	Child	L MFG	-3.06	0.003
				L SFG	-2.99	0.004
R MFG	2.83	0.049	Adult	L Hippocampus	3.96	< 0.001

Note: signs (-) indicate increases in functional connectivity concordant with age, such that negative signs indicate child-specific connectivity patterns and (implicit) positive signs indicate adult-specific connectivity. *F*-values and associated FDR-corrected *p*-values indicate ROI cluster-level effects. Height (connection-level) threshold: *p*_uncorrected_ < 0.01.

### 3.3 Nonsymbolic magnitude comparison

**Fig. 4:**
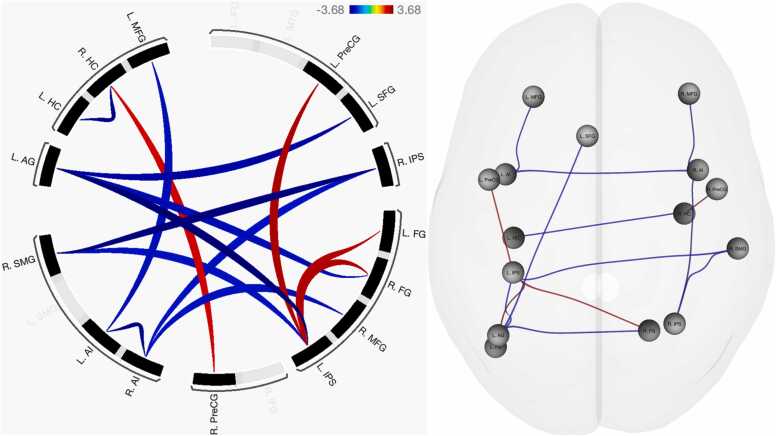
Functional connectivity differences associated with nonsymbolic magnitude comparison, controlled for general decision-making ability. Blue: increased FC negatively associated with age; red: increased FC positively associated with age.

Functional connectivity patterns were observed in nine seed regions (see Table 4): the left superior frontal gyrus, anterior insula, hippocampus, intraparietal sulcus, and fusiform gyrus; as well as the right anterior insula, hippocampus, fusiform gyrus, and supramarginal gyrus. See Fig. 4 for an overview of functional connectivity results.

**Table 4:** Functional connectivity patterns associated with nonsymbolic magnitude comparison.

Seed region	*F* (5, 83)	*p*_FDR_	Age group	Target region	*T* (87)	*p*_unc_
L SFG	6.04	0.001	Child	L Angular gyrus	-3.17	0.002
L Anterior insula	5.65	0.001	Child	R Anterior insula	-3.49	< 0.001
				L MFG	-2.94	0.004
R Anterior insula	5.53	0.001	Child	L Anterior insula	-3.49	< 0.001
				R MFG	-2.84	0.006
				R IPS	-2.65	0.010
L Hippocampus	4.59	0.005	Child	R Hippocampus	-3.40	0.001
L IPS	4.39	0.005	Adult	L Precentral gyrus	3.19	0.002
				R Fusiform gyrus	3.17	0.002
				L Fusiform gyrus	3.13	0.002
			Child	L Angular gyrus	-3.68	< 0.001
				R SMG	-2.65	0.009
R Hippocampus	4.21	0.006	Adult	R Precentral gyrus	2.78	0.007
			Child	L Hippocampus	-3.40	0.001
L Fusiform gyrus	3.69	0.010	Adult	L IPS	3.13	0.002
R Fusiform gyrus	3.67	0.010	Adult	L IPS	3.17	0.002
			Child	L Angular gyrus	-2.97	0.004
R SMG	2.78	0.048	Child	R IPS	-3.58	< 0.001
				L IPS	-2.65	0.009

Note: signs (-) indicate increases in functional connectivity concordant with age, such that negative signs indicate child-specific connectivity patterns and (implicit) positive signs indicate adult-specific connectivity. *F*-values and associated FDR-corrected *p*-values indicate ROI cluster-level effects. Height (connection-level) threshold: *p*_uncorrected_ < 0.01.

## 4 Discussion

The purpose of this study was to investigate the functional connectivity differences associated with children’s and adults’ performance of numerical discrimination tasks across the three representational formats outlined by the triple code model (TCM; e.g., Dehaene, 1992; Dehaene and Cohen, 1995): Arabic digits (e.g., “1”), number words (e.g., “two”), and nonsymbolic representations of numerical magnitude (e.g., •••). Using nineteen theoretically motivated regions of interest (ROI) commonly featured in the numerical cognition literature (cf. Faye et al., 2019; Iuculano et al., 2018; Moeller et al., 2015; Fias et al., 2013), as well as three regions for further exploration that did not produce statistically significant functional connectivity patterns (i.e., bilateral cerebellar lobule VI/VIIa and left anterior cingulate cortex), the current study is the first to empirically investigate developmental differences in functional connectivity across all three codes. Given that previous activity-based results indicated minor differences between children and adults in the recruitment of neural correlates across codes (Skagenholt et al., 2021), we hypothesized that neurocognitive differences between middle-school-aged children and young adults may be more apparent on the level of functional network connectivity (cf. Iuculano et al., 2018; Emerson and Cantlon, 2012). These results may be informative for the ongoing debate regarding the symbol-grounding problem in numerical cognition (e.g., Leibovich and Ansari, 2016), as the explicit comparison of age and code-dependent differences in functional number network connectivity could indicate a developmentally driven estrangement in the processing of number symbols and nonsymbolic numerical magnitude representations (Lyons et al., 2012). A major limitation of this study is that it did not include a comprehensive set of behavioral measures for both adults and children, which does not allow strong cognitive-behavioral inferences from the observed differences in functional connectivity patterns. The proposed interpretations, based on prior research within the scope of numerical cognition, should therefore be regarded as hypothetical inferences for further exploration in future research.

### 4.1 Functional number network connectivity in adults

Adults’ patterns of functional connectivity were largely consistent across both symbolic (i.e., Arabic and verbal) codes. Two functional connections were identical across both tasks, between the left intraparietal sulcus and hippocampus; and between the left hippocampus and right middle frontal gyrus. A partial overlap was observed for the right precentral gyrus node, connecting to the left hippocampus during Arabic digit comparison as opposed to the right hippocampus–more commonly associated with retrieval fluency (e.g., Menon, 2016)–during verbal number comparison. In contrast to the symbolic codes, developmental increases in functional connectivity associated with nonsymbolic magnitude processing were considerably sparser. Compared to children, adults demonstrated increased functional connectivity between the bilateral fusiform gyri and left IPS–indicative of a direct mapping of visual input onto the approximate number system (Iuculano et al., 2018)–and between the left IPS and precentral gyrus. Similar to the verbal code but distinct from Arabic digit processing, the right precentral gyrus was observed to connect to the right hippocampus, which may indicate a developmentally advantageous strategy of integrating numerical facts with the visual dot arrays maintained in working memory. Considering that the left precentral gyrus has been shown to be more prominently implicated in mental rotation and motor imagery tasks than its contralateral counterpart (Tomasino and Gremese, 2016), connectivity between this region and the left IPS may suggest a greater dependence on visuospatial processing with age.

In line with the prediction that symbolic number processing abilities gain independence from the approximate number system over developmental time, adults elicited unique functional connectivity between the left IPS and hippocampus. The left IPS has been argued to become tuned to symbolic number processing over developmental time (e.g., Vogel et al., 2015), which may indicate an automatization of processes underlying symbol recognition and magnitude mapping with age. This process appears to leverage declarative memory as supported by the default mode network’s hippocampal nodes (e.g., Nascimento Alves et al., 2019), together with a maintenance of numerical information in working memory, particularly as evidenced by connectivity between the left supramarginal gyrus and hippocampus during Arabic digit comparison. The left supramarginal gyrus has previously been characterized as the key correlate of overlap between the central executive and visuospatial components of working memory during children’s arithmetic problem solving (Metcalfe et al., 2013). In this vein, left hippocampus activation has been observed to increase during the maintenance of multiple items, suggesting that the region contributes not only to long-term but also working memory processing (Axmacher et al., 2007). The current pattern of adult left IPS–hippocampus–SMG connectivity may therefore indicate a developmentally driven functional integration of the working memory and central executive mechanisms required to efficiently maintain and interpret symbolic Arabic referents. This interpretation partially aligns with previous results (Skagerlund et al., 2018), showing that resting-state functional connectivity between the right IPS and left SMG is predictive of arithmetic ability in the same adult participant sample featured in the current study. The current indirect connectivity between the left IPS and SMG may be indicative of a similar but task-evoked left IPS specialization for symbolic Arabic numbers, in support of the same integration of working memory and central executive processes that aid arithmetic problem solving (Metcalfe et al., 2013). The lack of left SMG connectivity for the verbal code may imply that increased demands on the central executive and working memory are consistent for children and adults, given that written verbal representations of number occur less commonly in daily life, leading to overlapping (i.e., reduced developmental differences in) functional connectivity. However, connectivity between the left posterior MTG and right IPS during verbal number discrimination may fill a similar role. The MTG has been implicated as a key node in the semantic control network, crucial for accessing meaningful conceptual knowledge such as numerical values and their relative magnitude (Sommerauer et al., 2020), exhibiting interactions with the default mode and multiple demand (frontoparietal) networks (e.g., Davey et al., 2016). The interaction between these networks appears likely in adult verbal number comparison, where increased functional connectivity was observed between the right IPS and left MTG. This pattern indicates that the right IPS, as the primary neural correlate of the approximate number system and the “semantic hub” of number processing (e.g., Dehaene, 2011), may require retrieval of semantic numerical facts from the MTG as well as from the default mode network’s hippocampal formation (cf. Nascimento Alves et al., 2019).

Adults’ functional connectivity between the left IPS–hippocampus–right MFG, across both symbolic codes, may further indicate a developmentally driven differentiation of symbolic and nonsymbolic number processing. The right MFG has been specifically tied to numeracy as opposed to literacy (Koyama et al., 2017). The authors found that this right-hemisphere dominance for non-verbal processing aligned with the multiple demand (i.e., frontoparietal) network, which overlaps closely with the same integrative central executive and working memory network described above. The left hippocampus could therefore act as an intermediary hub node, connecting frontoparietal number network nodes with working memory and semantic retrieval capacities. Note that this pattern was consistent for both symbolic codes in adults, whereas children demonstrated increased connectivity to the left literacy-specific MFG (Koyama et al., 2017) during verbal number processing, in line with the proposal that different numerical representations become more integrated over developmental time (cf. developmental calculation model; Kaufmann et al., 2011). That is, the consistent left IPS–hippocampus–right MFG connectivity pattern for both symbolic codes may indicate that number words and Arabic digits are similarly perceived and manipulated *as numbers* due to increased exposure to number words and the development of an integrated white matter language–number semantic classification network common to both codes (Willmes et al., 2014).

Unique to Arabic digit comparison, the connectivity between the left hippocampus and right precentral gyrus is partially in line with previous research indicating that effective connectivity from a right SPL seed region to the right precentral gyrus and left SMG is predictive of arithmetic ability in children (Park et al., 2013). Given the developmental shift that specializes the left IPS for symbolic number processing, the observed pattern of left IPS, SMG, and right precentral gyrus connectivity through the left hippocampus may indicate a similar–but developmentally refined–connectivity pattern that supports foundational Arabic number discrimination abilities. The increase in right hippocampus to right precentral gyrus connectivity for the verbal code could indicate a functionally distinct integration of working memory and hippocampal memory retrieval. Beyond being implicated in motor planning and execution, the right premotor cortex–and precentral gyrus in particular–has been tied to the recollection of stimuli during working memory (e.g., n-back) tasks (Tomasino and Gremese, 2016). It stands to reason that written number words, due to their relative infrequency in naturalistic settings, place added emphasis on the maintenance of verbal information in working memory with concurrent retrieval of numerical facts as indicated by right IPS–right hippocampus connectivity. In comparison, Arabic digit processing may be automatized to such a high degree, in adults, that bilateral IPS connectivity to the right hippocampus suffices to retrieve numerical facts (e.g., [“two” = 2 = ••] > [“one” = 1 = •]).

### 4.2 Functional number network connectivity in children

Compared to adults, children’s unique increases in functional connectivity consistently targeted nodes of multiple, distributed functional networks. The recruitment of considerably more network nodes (13, 9, and 13 respectively for the Arabic, verbal, and nonsymbolic conditions compared to adults’ 7, 5, and 6) may indicate distributed, domain-general connectivity attributable to immature and inefficient cognitive strategies for number processing. It should be noted that such differences are not fully attributable to basic decision-making abilities in general, as the results of these analyses control for functional connectivity elicited by the basic letter case discrimination task. Differences in functional connectivity between children and adults may still be at least partially modulated by number-independent domain-general cognitive mechanisms, as evidenced by research indicating greater functional connectivity in children’s cingulo-opercular network during rest, as well as within their default mode and right frontoparietal networks during visual working memory task performance (Jiang et al., 2018). Future research should attempt to replicate the current results using additional cognitive-behavioral control measures of, minimally, visuospatial attention, working memory, and language ability (cf. Kaufmann et al., 2011).

In general, observed connectivity between frontoparietal (e.g., IPS, MFG, SFG), salience (e.g., insula, IFG), and default mode network (e.g., AG, hippocampus) nodes could indicate that children rely more on the frontoparietal network. The frontoparietal (task-positive) and default mode (task-negative) networks are anticorrelated, meaning that the activation of one network entails deactivation of the other by inhibition (Fox et al., 2005), as coordinated by the salience network (Menon and Uddin, 2010). Given that children performed the tasks slower than adults, that adults showed greater neural activity in default mode network nodes (e.g., cuneus, MTG, cingulate cortex; Skagenholt et al., 2021) linked to memory as opposed to attention demands (Smallwood et al., 2021), and prior research indicating children’s greater need for cognitive control and attention during number tasks (e.g., Ansari, 2008), this pattern may suggest inhibitory connectivity to the default mode network given increased use of frontoparietal (task-positive) resources.

In line with previous research on children’s arithmetic addition skills (Cho et al., 2012), increased functional connectivity between the bilateral hippocampus was observed across all tasks. The authors found that such a connectivity pattern showed positive correlations with arithmetic retrieval fluency. We suggest that the similar pattern, observed here, may indicate that children face increased demands on mapping numerical facts (subserved by the right hippocampus; Cho et al., 2012) to stimuli held in working memory (subserved by the left hippocampus; Axmacher et al., 2007).

Notably, children were observed to exclusively recruit nodes of the salience network (e.g., bilateral anterior insula) across tasks. The salience network has been observed to play a role in the habituation of recurring stimuli (e.g., Yamaguchi et al., 2004), which may indicate that children are more sensitized to numerical stimuli. As argued above, this could be a consequence of the frontoparietal number network inhibiting default mode network-mediated memory retrieval. This would also align with the right SMG connectivity observed across tasks, which is a key node in the bottom-up stimulus-elicited ventral frontoparietal attention network (e.g., Corbetta and Shulman, 2002). Activity in the right SMG is particularly related to unanticipated attentional shifts, driven by novel stimuli, implying that an increased reliance on the region could indicate children’s increased sensitization (potentially due to an increased need for visuospatial number line mapping) to numerical stimuli across representational formats. In a comparative sense, children’s functional connectivity to the right SMG could indicate *dyscalculia-like* behavior compared to adults (cf. Kaufmann et al., 2011), indicative of incomplete developmental maturation. This is moreover evident in nonsymbolic magnitude comparison, argued to be an evolutionarily hardwired capacity (e.g., Núñez, 2017), where children demonstrated similar and possibly immature increases in functional connectivity.

Two proposals can be made based on these results. First, similar (and likely inhibitory) salience and default mode network connectivity in children–regardless of the numerical representational format–may indicate an increased need to compare numerical stimuli against an abstract numerical magnitude representation, supported by the ANS and the activated representations’ overlapping position on the mental number line (i.e., • = 1 = one). Second, the consistency in adults’ connectivity patterns for the symbolic codes, but unique reliance on left precentral gyrus visuospatial functions in the nonsymbolic code, could indicate that developmental maturation leads numerical symbols to be understood in relation to other symbols retrieved from memory rather than explicit mental number line mapping (cf. Reynvoet and Sasanguie, 2016). Future research should particularly investigate the left IPS–hippocampus–right MFG connectivity pattern consistently identified for symbolic number processing in adults, and its relationship to (symbolic) arithmetic, ordinal number processing, and ANS-related (e.g., nonsymbolic discrimination acuity) abilities. In children, future research should attempt to replicate and control current results for additional domain-general cognitive measures not directly attributable to numeracy (e.g., working memory and attention).

## 5 Conclusions

Middle-school-aged children and young adults, while recruiting a similar set of neural substrates during the processing of numerical magnitude across numerical representational codes (cf. Skagenholt et al., 2021), differ in terms of the neural circuits employed for such tasks. Adults were observed to elicit fewer but more consistent patterns of connectivity across the symbolic codes. The current results question the primacy of the approximate number system (ANS) in adult-level number discrimination, as it appears that symbolic numbers may rather be understood in reference to semantic memory-based numerical facts (e.g., Reynvoet and Sasanguie, 2016). Conversely, children’s functional connectivity across tasks suggests frontoparietal network dominance, likely providing inhibitory connectivity in default mode and salience network nodes. This pattern of results indicates that children may require the mapping of both symbolic and nonsymbolic numerical referents to an amodal and abstract magnitude representation, while developmental maturation could serve two main effects: a decoupling of the functional circuitry supporting symbolic (memory-based) and nonsymbolic (visuospatial) number processing; and the establishment of integrated circuits for retrieval, working memory, and visuospatial processes for format-independent number processing (Kaufmann et al., 2011). Moreover, these results could be of explanatory value for reported absences of transfer effects between symbolic arithmetic ability and ANS acuity in adults (Lindskog et al., 2016), implying that symbolic and nonsymbolic numerical cognition may become decoupled over developmental time. While the current results describe connectivity patterns between theoretically motivated regions and provide hypotheses for their cognitive-behavioral implications, future research should attempt to broaden the scope of developmental connectivity differences in number processing. For instance, future research could leverage whole-brain (e.g., multi-voxel pattern analysis) techniques, longitudinal neuroimaging paradigms over the course of typical development, and concrete associations between functional connectivity patterns and behavioral outcome measures.

## Declaration of Competing Interest

The authors declare that they have no known competing financial interests or personal relationships that could have appeared to influence the work reported in this paper.

## Data Availability

Associated group-level neuroimaging data is available from the Open Science Foundation: https://osf.io/n5uvy/?view_only=30d349bd1c5943c98929b180f81a873c.
